# Characteristic Analysis and Design Optimization of Bubble Artificial Muscles

**DOI:** 10.1089/soro.2019.0157

**Published:** 2021-04-16

**Authors:** Richard Suphapol Diteesawat, Tim Helps, Majid Taghavi, Jonathan Rossiter

**Affiliations:** ^1^ Department of Engineering Mathematics, University of Bristol, Bristol, United Kingdom.; ^2^ Bristol Robotics Laboratory, Bristol, United Kingdom.

**Keywords:** wearable, actuator, pneumatic, fluidic, artificial muscle

## Abstract

Soft robotics requires new actuators and artificial muscles that are lighter, less expensive, and more effective than current technologies. Recently developed bubble artificial muscles (BAMs) are lightweight, flexible, inexpensive, pneumatic actuators with the capability of being scalable, contracting at a low pressure, and generating sufficient tension and contraction for assisting human mobility. The BAMs are simply fabricated by using a commercial plastic tubing with retaining rings, forming a “bubble” shape and creating a series of contractile units to attain a desired stroke. They can deliver high contraction through optimization of actuator length and radius, or high tension by strengthening their materials to operate at high pressure. Here, we present a detailed analysis of BAMs, define a model for their actuation, and verify the model through a series of experiments with fabricated BAM actuators. In tests, a maximum contraction of 43.1% and a maximum stress of 0.894 MPa were achieved, corresponding to the BAM lifting a load 1000 times its own weight (5.39 g). The BAM model was built to predict experimental performance, for example, the relationship between tension and contraction at various applied pressures, and between contraction and pressure. Characteristic analysis and design optimization of the BAM are presented as an approach to design and manufacture the ideal “bubble” actuator at any required dimensions. A BAM orthosis is demonstrated as assisting a sit-to-stand transition on a leg mechanism, constructed to match the scale of a human's lower limb. Guidelines for further improvement of the BAM are also included.

## Introduction

Assistive technologies have emerged to endow humans with more capabilities and independence in life, especially among older adults. Powered exoskeletons and orthoses have considerably developed over the past decades to provide wearers with more strength and mobility.^1^ These rigid exoskeletons can be divided into two categories based on their purposes: (1) enabling and restoring mobility in patients with disabilities and (2) strengthening workers and soldiers to undertake heavy activities for industrial or military applications.^2^ However, there are many ongoing challenges and requirements, including low weight, flexibility, comfort and adaptability to human body, esthetics, affordability, long lifetime, reliability, and safety. In addition, restriction of natural body motion and damage to the body of healthy individuals must be avoided.

Soft robots have advantages over conventional rigid robots thanks to their light weight and compliance, being more suitable for direct interactions with the human body while potentially reducing harm. Pneumatic artificial muscles (PAMs) are one of the most commonly used actuators to drive assistive soft exoskeletons.^3^ Although pneumatic exoskeletons were initially made of mostly rigid components,^4,5^ more recently they have been developed with more flexible, compliant elements.^6–15^ Soft exoskeletons and artificial muscles have also used a range of power sources, including cable-tendon-driven mechanisms, that is, Exosuit,^16^ Myosuit,^17^and XoSoft^18^; direct electro-mechanical energy transduction in polymers, for example, polyvinyl chloride (PVC) gel,^19,20^ dielectric elastomers,^21,22^ dielectrophoretic liquid zipping actuators,^23^ and Peano-HASEL^24^; and thermo-mechanical actuation such as coiled polymer.^25^

PAMs are soft, flexible contractile actuators that change shape and contract when activated by pressurized air.^26^ The McKibben pneumatic muscle is widely used in soft exoskeletons. It is made of a length of an elastic tubing enclosed by a braided sleeve, which contracts to form a cylindrical shape under an applied pressure.^27^ Similarly, straight fiber PAMs use an elastic tube featuring reinforced fibers to increase membrane strength, and they form a spherical shape when actuated.^28–31^

Other soft pneumatic actuators (SPAs) apply this concept of using a stiff embedded sheet on the inside of soft elastomer^32^ or creating a three-dimensional (3D)-printed soft contractile actuator consisting of stiff and soft composites.^33^ These PAMs use an elastic material as the actuator's membrane, which stretches and expands under high applied pressure, and use a stiffer material to limit membrane expansion, resulting in a pre-determined shape and contraction.

On the other hand, pleated pneumatic artificial muscles (PPAMs) only use a high-strength inelastic material as an actuator membrane constrained by special end fittings to create equal radial pleats at the actuator ends, contracting to an elliptical shape under pressure.^34^ The PPAMs were initially used as an artificial muscle for a gripper, a robot arm, and a walking or running mechanism.^35^ Pouch motors use a commercial flexible plastic material to create a lightweight series contractile actuator, allowing low-pressure actuation.^36^ Bending SPAs, made of a thin-walled flexible tubing, have achieved large bending motions and high torque at low pressure operation.^37^ These three actuators benefit from their inelastic material's strength to operate under pressure and produce contraction and tensile force.

Recently, new contractile PAMs made of a commercial inelastic plastic tubing with the addition of soft rubber rings (series pneumatic artificial muscle—sPAM) or rigid metal rings (bubble artificial muscles—BAMs) have been presented. The sPAM^38^ was developed for navigation and survey applications, enabling a low-pressure soft continuum robot to steer and operate in constrained and cluttered environments,^39^ whereas the BAM was developed as a human-like muscle to assist human mobility, for example, for aiding knee flexion during walking.^40^

**FIG. 1.**
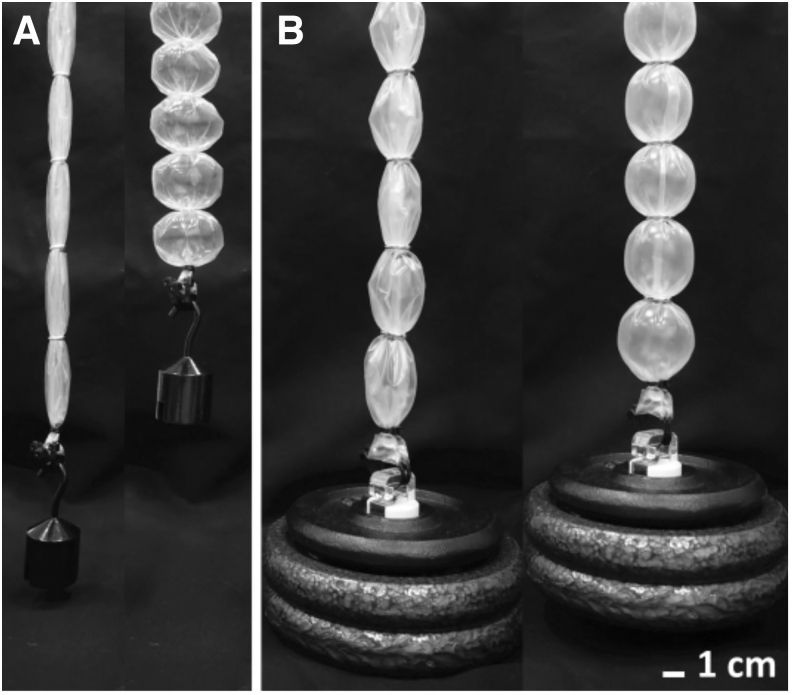
Actuation of BAMs. **(A)** A BAM made of thin material (30.0 μm) delivers high contraction of 37.0% while lifting 0.5 kg. **(B)** A BAM made of thicker material (125.0 μm) delivers high tensile force, lifting 3.0 kg at a contraction of 10.2%. BAMs, bubble artificial muscles.

The BAMs are lightweight, compliant, and inexpensive pneumatic muscles, designed to have similar structure and function as the PPAM while being considerably simpler, less expensive, and lower weight; being made of a thin, flexible inelastic tubing and stiff retaining rings. This grants the BAM flexibility and low- to high-pressure actuation to deliver either high contraction or high tensile force depending on the thickness and stiffness of the tubing material (Fig. 1; Supplementary Movie S1). 

The BAM can be designed to deliver the most suitable mechanical assistance for different parts of the human body, which requires different tensile forces and amounts of contraction. To enable this design flexibility, we present characteristic analysis and design optimization of BAMs, develop an actuator model encompassing the unique buckled folds at the rings, and verify the model against experimentation with a range of BAM actuators.

## Pneumatic Actuators

### Pleated pneumatic artificial muscle

We first consider the PPAM^34^ (Supplementary Fig. S1A), from which models of the sPAM and the BAM are derived. The characteristics of the PPAM are based on actuator length *L*, actuator radius *R*, and applied pressure *P*, with the assumption of inelastic material behavior. The PPAM mathematical model was derived by using an elliptical integral with *m* and *φ_R_* as dominant parameters to determine the actuator shape at any contraction. Three main equations are used to calculate the contraction *c* and tensile force *T* of the PPAM, as follows:

(1) $$L=\frac{R}{\sqrt{m}\cos\varphi_{R}}F\left(\varphi_{R}∕m\right)$$

(2) $$c=1-\frac{2R}{L}\left(\frac{E\left(\varphi_{R}∕m\right)-\frac{1}{2}F\left(\varphi_{R}∕m\right)}{\sqrt{m}\cos\varphi_{R}}\right)$$

(3) $$T=\pi PR^{2}\frac{1-2m}{2m\cos^{2}\varphi_{R}}$$

*m* defines the actuator's shape and its contraction, where zero contraction or maximum contraction are reached when *m* is equal to 0 or 0.5, respectively (0≤m≤0.5), as shown in Supplementary Figure S1B. $\varphi_{R}$ is a characteristic angle mathematically related to the shape of the actuator membrane $\left(0\leq \varphi_{R}\leq \pi ∕2\right)$, calculated from Equation (1) when substituting an *m* value, and actuator size, *L* and *R*. This results in a $\varphi_{R}$ value for each *m* value; $F\left(\varphi_{R}∕m\right)$ and $E\left(\varphi_{R}∕m\right)$ are the elliptic integrals of the first kind and second kind, respectively (Elliptic Integral section in Supplementary Data). *c* and *T* can then be calculated by Equations (2) and (3) respectively, given an applied pressure *P*. Full details related to the PPAM model can be found in the Pleated Pneumatic Artificial Muscles section in Supplementary Data.

### Series pneumatic artificial muscle

The sPAM is made of a long plastic tubing and rubber O-rings, creating a series of pneumatic actuators similar to the PPAM.^38^ Unlike the PPAM, an inactive region appears in the sPAM when the actuator length of the sPAM is greater than a certain value. This occurs when the actuator radius reaches the maximum material radius (the radius of the plastic tubing used to build the sPAM), causing the actuator to form a cylindrical region in the middle of the actuator, preventing the generation of further contraction, and thus limiting the overall contraction ratio. Although each contractile unit of the sPAM generates high contraction close to that of the PPAM model, it produces much lower tensile force than the PPAM due to its low material strength, which limits applied pressure.

### Bubble artificial muscle

**FIG. 2.**
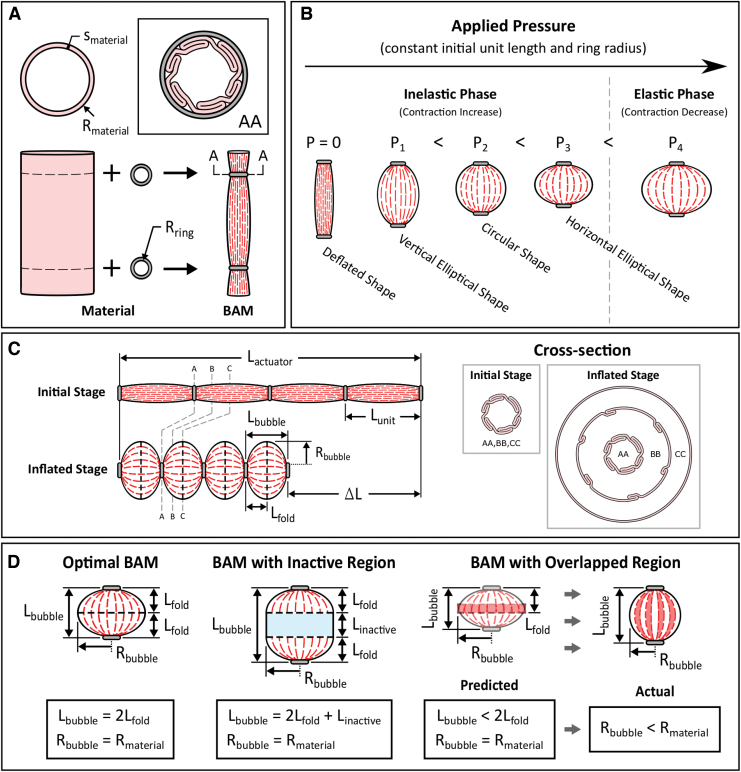
**(A)** Components of a single-unit BAM: plastic tubing and two metal rings, and the cross-section of the BAM at the actuator end (AA). **(B)** BAMs at different applied pressure, demonstrating the inelastic and elastic phases. **(C)** Definitions of BAM parameters, images showing an optimal BAM at the initial stage and the inflated stage, and the cross-section of the actuator of these two stages at different points along the actuator (AA, BB, and CC). **(D)** Different expanded shapes of different single-unit BAMs at maximum applied pressure in the inelastic phase illustrating Lbubble and Rbubble at maximum shape expansion of (*left*) an optimal BAM, (*middle*) a BAM with an inactive region (*blue area*), and (*right*) a BAM with an overlapped region (*red area*). Color images are available online.

The BAM was developed to deliver high tensile force while maintaining high contraction. This is achieved by introducing stiff retaining rings and strong actuator material, allowing the BAM to sustain high pressure. The BAM is made by using a polyethene plastic tubing and metal retaining rings (Fig. 2A). Two metal rings (gray in Fig. 2A) are inserted along the plastic tubing (pink in Fig. 2A) to create a single contractile unit. These metal rings constrain the tubing to form a tight folded shape within the ring radius (cross-section AA in Fig. 2A). Non-uniform folding of the plastic tubing extends along the actuator in the axial direction (red dashed lines in Fig. 2A). These induced folds function similarly to the pleats in the PPAM. The result is a PAM that is considerably less expensive and easier to manufacture compared with the PPAM, while exhibiting higher tensile force compared with the sPAM.

When inflating the BAM by pressurized air, the actuator membrane unfolds and expands radially, and thus the actuator contracts, forming different expanded shapes, that is, a vertical elliptical shape, a circular shape, and a horizontal elliptical shape, depending on the level of applied pressure *P* (Fig. 2B). Increasing the applied pressure results in an increase in actuator contraction. This increase happens only in the inelastic phase, whereby the membrane flexes and the actuator changes shape, with negligible elastic stretching of the membrane. If the pressure is increased beyond the point of maximum contraction, behavior enters an elastic phase.

In the inelastic phase (Fig. 2B), increasing the applied pressure causes the actuator membrane to stretch, inflating like a balloon, which results in a decrease in contraction. The optimal contraction happens when the actuator forms a horizontal elliptical shape in the inelastic phase, which resembles a “bubble,” leading to the name of the actuator, “Bubble Artificial Muscle.”

As shown in Figure 2A, the BAM is fabricated from a plastic tubing with a maximum material radius $R_{material}$ and a material thickness $s_{material}$ and metal rings with ring radius $R_{ring}$. With this design and fabrication method, the BAM can comprise many contractile units aligned in series as demonstrated in Figure 2C, enabling it to achieve any desired total strokes. For example, the BAM in Figure 2C consists of four contractile units; therefore, it can produce a maximum stroke four times that of a single unit. At the initial stage, the length of the entire BAM series of actuators is defined as an initial actuator length $L_{actuator}$, whereas that of each contractile unit is defined as an initial unit length $L_{unit}$. When inflated, the BAM contracts by stroke ΔL and a total contraction *c*, where $c=\Delta L∕L_{actuator}$. Its inflated unit length and inflated unit radius are defined as a bubble length $L_{bubble}$ and a bubble radius $R_{bubble}$ at maximum shape expansion (*m* = 0.5), respectively.

The optimal contraction $c_{optimal}$ can be delivered by a BAM having $L_{unit}$ equal to the optimal unit length $L_{optimal}$, actuated at the maximum applied pressure that retains inelastic behavior (resulting in maximum shape expansion). At any applied pressure *P*, a folded membrane around the metal rings always exists, forming an actuator shape as in cross-section AA in Figure 2C. However, when the optimal BAM is inflated, the amount of folded membrane is reduced incrementally with distance from the metal rings as can be seen in cross-sections AA, BB, and CC.

A fold length $L_{fold}$ describes the length of the folded region of the BAM (red dashed lines) at maximum shape expansion in the axial direction, beginning from the actuator end and ending at the point where no folding appears on the membrane surface, shown as a black dashed line. Therefore, for the optimal BAM, $L_{fold}$ is equal to 0.5 of $L_{bubble}$ (or $L_{bubble}=2L_{fold}$) as shown in Figure 2D (left). $L_{fold}$ varies depended on the ring radius $R_{ring}$, material radius $R_{material}$, and material thickness $s_{material}$, causing a different amount of folding at the actuator ends.

Besides the optimal BAM, other expanded shapes can be obtained at the maximum applied pressure, depending on $L_{unit}$, $R_{ring}$, and $R_{material}$. For example, when an inactive region occurs, $L_{inactive}$ defines the axial length of the inactive region (blue in Fig. 2D, middle), and $L_{fold}$ is the same as that of the optimal BAM; therefore, $L_{bubble}=2L_{fold}+L_{inactive}$. Alternatively, for shorter unit lengths, an overlapped region (red in Fig. 2D, right) occurs due to the crossover of folding from both actuator ends ($L_{bubble,predicted}<2L_{fold}$). This results in an effectively stiffer actuator membrane, leading to partial shape expansion at the maximum applied pressure ($R_{bubble,actual}<R_{material}$) and lower contraction ($L_{bubble,actual}>L_{bubble,predicted}$).

The BAMs are designed to produce sufficiently high force and contraction to drive an orthotic to assist human muscles. As described earlier, BAM characteristics depend on an initial unit length $L_{unit}$, a ring radius $R_{ring}$, an applied pressure *P*, and a material thickness $s_{material}$. For example, the optimal “bubble” shape expansion and high contraction can be attained by carefully selecting $L_{unit}$ and $R_{ring}$ to avoid an overlapped region or an inactive region, whereas high force generation can be achieved by increasing $s_{material}$ and maximizing *P*. Varying these four parameters leads to different performance of the BAM.

## Experimental Setup

Several experiments were conducted to investigate the effects of *P*, $L_{unit}$, $R_{ring}$, and $s_{material}$ to evaluate the resulting BAM performance. Three commercially available low-density polyethylene layflat tubes (Young's Modulus *E* = 0.3 GPa) with different thicknesses ($s_{material}=$ 30.0, 62.5 and 125.0 μm) and similar radii (∼17 mm) were selected for fabrication into BAMs as shown in Supplementary Table S1. The metal rings were made of metal with a thickness of 1.30 mm and an internal radius of 4.5 mm.

To evaluate the BAM performance, two types of experiments were undertaken: an isometric test and an isotonic test. For isometric testing, the BAM was oriented vertically, with the top end mounted on an acrylic frame connected to a 1 kN load cell (700 Series S Beam Load Cell; Load Cell Shop, UK) and the bottom end attached to a linear actuator (LACT8P; Concentric International, USA). The tensile force was measured by the load cell through a load cell amplifier (RW-ST01A; SMOWO, China), and the actuator stroke was controlled by the linear actuator and recorded by a laser displacement sensor (LK-G152; Keyence, Japan). Pressurized air was supplied by an air compressor (CW 100/24 AL; Werther International S.p.A., Germany) connected to the actuator through a pressure regulator (AR20-F02H010B; SMC, UK) and a solenoid valve (WZ-98302-46; Cole-Parmer, UK), which were used to regulate the pressure level and inflate and deflate the actuator. A pressure sensor (HSCDANN030PGAA5; Honeywell, USA) was located close to the actuator to measure the applied pressure.

For isotonic testing, the bottom end of the actuator was disconnected from the linear actuator and a test mass was hung instead. The rest of the test environment was identical to the isometric test.

All tested BAMs consisted of four contractile units, excluding the optimal-unit-length experiment. Isometric tests were used to investigate the effects of varying *P*, $L_{unit}$, and $R_{ring}$ of BAMs with the same $s_{material}$, and the relationship between tensile force and pressure, and between tensile force and contraction, of BAMs with different $s_{material}$. Isotonic tests were used to investigate the maximum contraction of BAMs while holding external loads. In the experimental results that follow, maximum contraction $c_{max}$ is the contraction at zero tensile force (T=0) and maximum tensile force $T_{max}$ is the tensile force at zero stroke (ΔL=0).

## Results

### Increasing applied pressure (*P*)

**FIG. 3.**
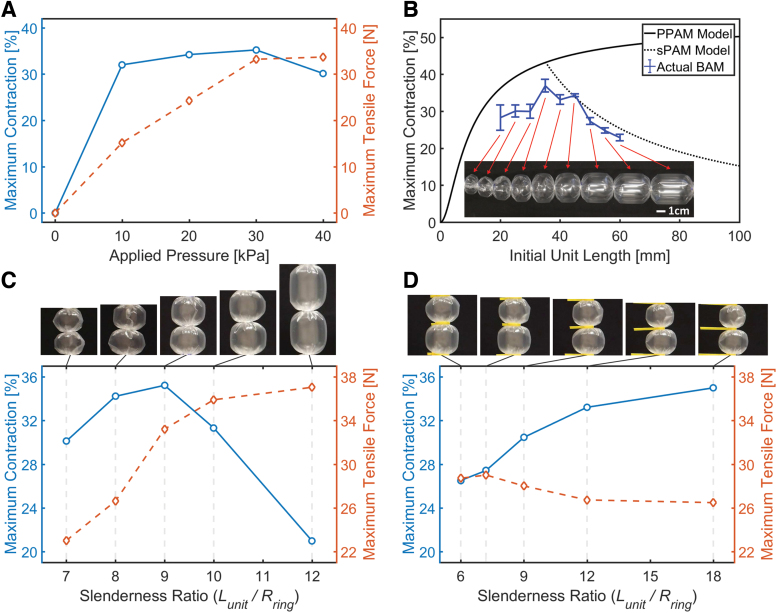
**(A)** Maximum contraction (*left y*-axis) and maximum tensile force (*right y*-axis) of a BAM with Lunit = 40.5 mm, Rring = 4.5 mm, and smaterial = 62.5 μm, operated at different *P*. **(B)** Maximum contraction of a series of BAMs with different Lunit but constant Rring = 4.5 mm and smaterial = 30.0 μm, actuated at *P* = 10.0 kPa (the PPAM and sPAM models are plotted, and the image of the actual BAMs is *inset*). **(C, D)** Maximum contraction (*left y*-axis) and maximum tensile force (*right y*-axis) of the BAMs made of smaterial = 62.5 μm with different slenderness ratios *SR* (Lunit/Rring), where **(C)** Rring = 4.5 mm and **(D)** Lunit = 40.5 mm, actuated at *P* = 30.0 kPa (images show the maximum expanded shape of the middle two contractile units of the tested BAMs). PPAM, pleated pneumatic artificial muscle; sPAM, series pneumatic artificial muscle. Color images are available online.

A BAM with $L_{unit}=40.5mm$, $R_{ring}=4.5mm$, and $s_{material}=$ 62.5 μm is considered here as an example to show the effect of increasing *P*. As shown in Figure 3A, the BAM achieves the maximum contraction of 32.0%, 34.2%, and 35.2% at P= 10.0, 20.0, and 30.0 kPa, respectively, with maximum contraction decreasing to 30.1% at P= 40.0 kPa.

This result can be explained by the progression from the inelastic phase to elastic phase, as presented in Figure 2B. Increasing applied pressure over a certain threshold (between 30 and 40 kPa in this case) causes the actuator membrane to stretch (behavior enters the elastic phase) and results in a reduction in the maximum contraction. Further increasing pressure would lead to irrecoverable plastic deformation and ultimately rupturing, therefore experiments were halted when elastic behavior occurred, before rupture. This stretching behavior can occur at any contraction when exceeding the pressure threshold (Supplementary Fig. S3A).

### Varying initial unit length ($L_{unit}$)

Five BAMs with $L_{unit}$ of 31.5, 36.0, 40.5, 45.0, and 54.0 mm but constant $R_{ring}$ of 4.5 mm (slenderness ratio $SR=L_{unit}∕R_{ring}=$ 7, 8, 9, 10 and 12) and $s_{material}$ of 62.5 μm were tested to demonstrate the effect of varying $L_{unit}$. Constant *P* of 30 kPa was used to inflate the BAMs without stretching behavior.

As shown in Figure 3C, the greatest maximum contraction occurs at $L_{unit}=$ 40.5 mm (SR= 9). Lower maximum contraction appears at shorter $L_{unit}$ (SR= 7 and 8) and longer $L_{unit}$ (SR= 10 and 12) due to the appearance of the overlapped region and the inactive region, respectively. In contrast, the maximum tensile force increases when $L_{unit}$ is higher. This is because the BAM has larger membrane surface area at ΔL= 0 (membrane surface area is roughly equal to $2\pi R_{ring}L_{unit}$). The fluid pressure is applied over a larger surface, resulting in higher total radial force and thus higher tensile force. Further results at other stroke ranges can be found in Supplementary Figure S3.

### Varying ring radius ($R_{ring}$)

A BAM with constant $L_{unit}$ of 40.5 mm and $s_{material}$ of 62.5 μm was created by using plastic cable ties in place of metal rings due to the ease in controlling internal radius. Ring radii of 6.750, 5.625, 4.500, 3.375, and 2.250 mm (SR= 6, 7.2, 9, 12, and 18) were selected to investigate the effect of varying $R_{ring}$. The actuators were inflated at constant P= 30 kPa.

From Figure 3D, decreasing $R_{ring}$ results in higher maximum contraction and reduction of the inactive region. However, an overlapped region occurs when the BAM has too small $R_{ring}$, causing lower contraction than predicted by the PPAM model. As in the prior length-varying tests (Fig. 3C), the maximum tensile force increases with $R_{ring}$ because of the larger membrane surface area at ΔL=0.

### Optimal unit length ($L_{optimal}$)

The effects of the overlapped region and the inactive region, which influence $L_{optimal}$, were studied further with the BAM made of the thinnest material. A series of BAM units with different $L_{unit}$ from 20.0 to 60.0 mm at increments of 5.0 mm were built as a single long actuator. Metal rings with $R_{ring}=$ 4.5 mm and a thin plastic tubing with $s_{material}=$ 30.0 μm were selected for fabrication. $L_{optimal}$ was calculated as 35.2 mm (see BAM Optimal Unit Length and Optimal Ring Radius section in Supplementary Data). The experimental procedure involved inflating the BAM at P= 10 kPa, measuring its inflated length (using a calliper), and deflating. This experiment was repeated three times for each $L_{unit}$, and no external load was applied on the actuator. The maximum measured contraction of each single-unit BAM is presented in Figure 3B.

The largest contraction (mean at 36.9% and maximum at 38.7%) was observed for the BAM with $L_{unit}=$ 35.0 mm, which is close to the predicted $L_{optimal}$ (35.2 mm). The BAMs with $L_{unit}<L_{optimal}$ follow the general trend of the PPAM model,^34^ but there is an offset (a reduction in contraction) as a result of the overlapped membrane. The BAMs with $L_{unit}>L_{optimal}$ follow the general trend of the sPAM model.^38^

### Varying material thickness ($s_{material}$)

**FIG. 4.**
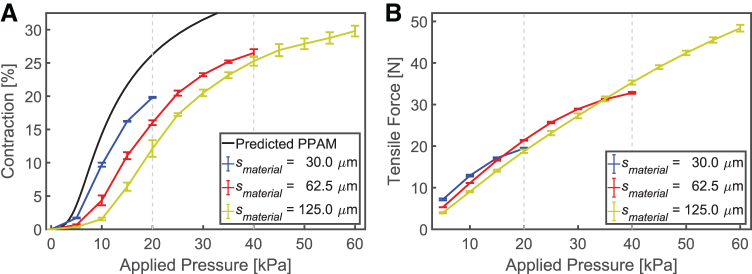
Contraction and tensile force of BAMs (Lunit = 40.5 mm and Rring = 4.5 mm) with three different smaterial undergoing **(A)** isotonic testing while with a 1 kg load and **(B)** isometric testing with Δ*L* = 0 mm; operated from 0 kPa to maximum pressures of 20, 40, and 60 kPa, respectively. The two *vertical gray dashed lines* indicate the maximum tested pressure of the BAMs made from 30 and 62.5 μm thick material, respectively. Color images are available online.

Isotonic and isometric tests were performed to evaluate the BAM's capability to produce contraction with an external load of 1 kg and maximum tensile force at ΔL= 0 mm, respectively. The experimental results for BAMs with the same $L_{unit}=$ 40.5 mm and $R_{ring}=$ 4.5 mm but different $s_{material}=$ 30.0, 62.5, and 125.0 μm are presented in Figure 4. The predicted PPAM contraction (which neglects the thickness of the actuator membrane) is included in Figure 4A: The PPAM model predicts infinite tensile force at zero contraction, so this is not included in Figure 4B.

Overall, at the same applied pressure, BAMs with thicker membranes produce less contraction and generally less tensile force, deviating increasingly from the PPAM model prediction (Fig. 4A). From Figure 4B, the tensile force of each BAM tends to decrease due to stretching behavior when applied pressure approaches their maximum pressure; increasing applied pressure further can cause the BAM to burst. The results of the isometric test measuring tensile force of the three BAMs at strokes other than zero are shown in Supplementary Figure S4.

**FIG. 5.**
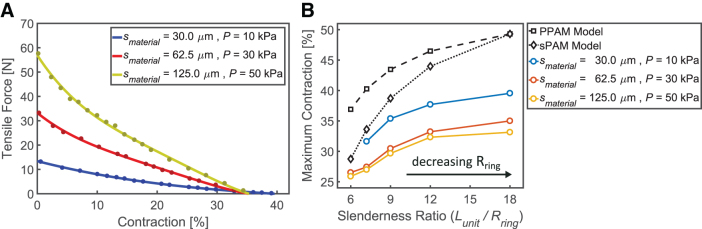
**(A)** The relationship between tensile force and contraction for BAMs with different smaterial but the same Lunit = 40.5 mm and Rring = 4.5 mm, operated with different applied pressure *P*. *Dots* indicate measured data from experiments, to which curve-fitting lines have been added. **(B)** Maximum contraction of BAMs made by using cable ties with Lunit = 40.5 mm, with different slenderness ratios *SR* (Lunit/Rring), Rring, and smaterial, actuated at different *P*. PPAM model (*dashed line*) and sPAM model (*dotted line*) predictions are also shown. Color images are available online.

Further isometric tests were performed to obtain the relationship between tensile force and contraction of BAMs with different $s_{material}$, as shown in Figure 5A. They were tested with different *P* of 10, 30, and 50 kPa, based on their $s_{material}$, well below the maximum pressures observed in Figure 4. As a result, the thin-membrane BAM can produce the highest contraction of 39.5%, and the thick-membrane BAM can generate the highest tensile force up to 56.9 N.

Figure 5B shows the effect of decreasing $R_{ring}$ on maximum contraction for BAMs made from three different $s_{material}$. BAMs made from $L_{unit}$ = 40.5 mm and $R_{ring}$ = 6.750, 5.625, 4.500, 3.375, and 2.250 mm were evaluated under zero tensile load. *P* was chosen to be well below the maximum pressure of each material. The theoretical maximum contractions of the PPAM and sPAM are also shown and are listed in Supplementary Table S2.

As can be seen in Figure 5B, discrepancy between the PPAM and sPAM models is maximized at high $R_{ring}$ due to the inactive region. Decreasing $R_{ring}$ reduces the size of the inactive region, and the sPAM model more closely matches the PPAM model. Decreasing $R_{ring}$ increases the maximum contraction of the actual BAMs. However, it also results in a higher reduction in BAM contraction compared with the sPAM model due to the material thickness (both the PPAM and sPAM models assume a zero-thickness membrane). This behavior occurs with all $s_{material}$ but is more pronounced with higher $s_{material}$. The sPAM model includes only the effect of the inactive region, but higher fold lengths, and a consequential overlapped region, are more likely to appear in actual BAMs with higher $s_{material}$. Consequently, to more accurately capture BAM actuation we must include these effects in the model.

## Discussion

### BAM performance

Typical pneumatic actuators can deliver a maximum contraction of around 25–35%, for example, McKibben muscle and Pouch Motor.^26,36^ Since the maximum expanded shape of the PPAM, sPAM, and BAM actuators is the horizontal ellipse (Fig. 2B), they can all feasibly reach a maximum contraction of 45.5% (Ref.^35^). A single-contractile-unit PPAM with $L_{actuator}=$ 100.0 mm, $R_{actuator}=$ 12.5 mm and weight of 58.3 g was able to deliver a maximum contraction of 41.5% and a maximum tension of 3500 N under P= 300 kPa.^34^ An sPAM with $R_{ring}=$ 2.5 mm delivered a maximum contraction and tension of ∼40% and 9 N, respectively, actuated at P= 10.34 kPa.^38^

Compared with these PAMs, the BAM can be designed to deliver either high contraction when using the thinnest $s_{material}$ and the smallest $R_{ring}$ or high tension when using the thickest $s_{material}$ and operating under high *P*. The highest contraction of 43.1% was delivered by the BAM with $s_{material}=$ 30.0 μm, $L_{unit}=$ 40.5 mm, $R_{ring}$ = 2.0 mm, and an actuator weight of 2.83 g while producing a maximum tensile force of 13.2 N.

Similarly, a BAM with $L_{unit}=$ 40.5 mm, $R_{ring}$ = 4.5 mm, and higher $s_{material}$ of 125.0 μm delivered a maximum tensile force of 56.9 N ( = 0.894 MPa), which corresponds to lifting a load 1000 times its own weight (5.39 g), and delivers a maximum contraction of 35.2% when operated at a pressure of P= 50.0 kPa (Fig. 5A). Its weight is less than one tenth that of the PPAM because of its simpler actuator ends and lighter materials. Three BAMs made of the same actuator design and materials had a similar tension–contraction relationship; the sample variation is shown in Supplementary Figure S5.

### Comparison between PPAM, sPAM, and BAM

**FIG. 6.**
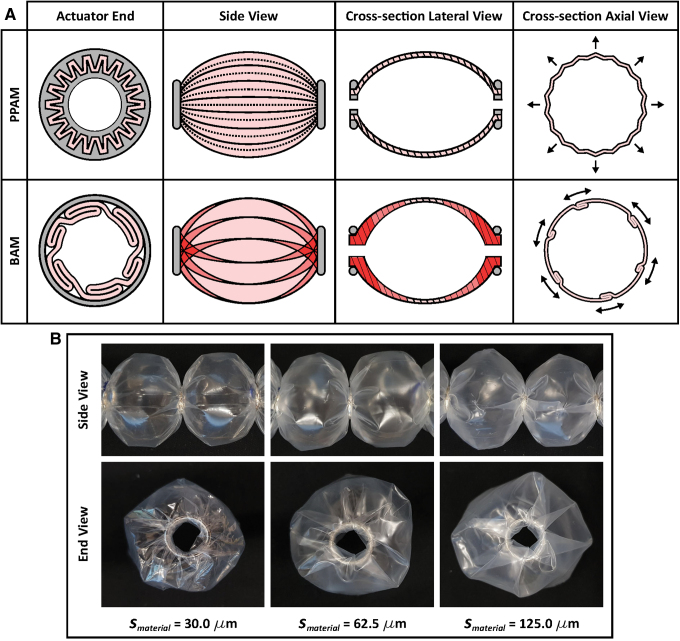
**(A)** Cross-section view of the actuator end, side view, cross-section lateral view, and cross-section axial view showing membrane expansion, comparing the PPAM and BAM. The *pink area* is the actuator's material, and the *gray area* is the actuator ends: the special end fitting for the PPAM and the metal ring for the BAM. The *pink color* indicates thin membrane, whereas the *red color* indicates where multiple membrane folds occur. **(B)** Actuator side and end view of BAMs with different smaterial of 30.0, 62.5, and 125.0 μm, but the same Lunit = 40.5 mm and Rring = 4.5 mm. As material thickness is increased, the increase in fold length is clearly visible. Color images are available online.

Although the BAM superficially resembles the PPAM, they have different fundamental structures due to different actuator ends: a special end fitting for the PPAM and a metal ring for the BAM (Fig. 6A). The PPAM has uniform pleats in the radial direction, whereas the folds of the BAM are in the lateral direction. These lateral folds are freely and nonuniformly folded around the metal ring, creating a region of overlapping folds, which naturally resists the radial expansion of the BAM. As membrane thickness increases, the amount of lateral folds also increases (Fig. 6B), increasing resistance to BAM shape expansion and reducing contraction and tension. However, higher thickness materials can withstand higher applied pressures, leading to higher expanding force and thus higher tensile force and maximum contraction of the BAM when loaded (Fig. 4).

When the BAM is inflated, the folds unfold by sliding and bending to expand the actuator shape (Fig. 6A), approaching the circular actuator cross-section (CC in Fig. 2C). The thickness of the folded membrane along the actuator axis *x* may be defined as the bubble surface thickness $s_{bubble}\left(x_{i}\right)$, where *x_i_* is the distance from the actuator center (Supplementary Fig. S1). $s_{bubble}\left(x_{0}\right)$ is highest at the actuator ends (higher than $s_{material}$), where many overlapping folds occur, and it reduces toward the middle of the actuator where no overlapping folds occur such that $s_{bubble}\left(x_{n}\right)=s_{material}$ when inflated (Fig. 6A). Unlike the BAM, the PPAM has no overlapping folds due to its radial folding structure, resulting in zero-friction shape expansion when inflated.

Although the sPAM and BAM share some functionality, the sPAM was designed to control the movement of lightweight continuum robots. In contrast, the BAM was designed as a high-power artificial muscle (e.g., for wearable assistive applications), necessitating higher contraction and tension. Various aspects of the BAM design result from these higher required performance metrics (high-thickness plastic tubing that allows for considerably higher applied pressure, metal retaining rings that withstand high radial force and folding analysis).

An isotonic test using a load of 1 kg demonstrates the difference in performance between these two actuators (Supplementary Fig. S6). With the BAM, the metal rings maintained an effective actuator shape, resulting in the BAM contraction after theoretical PPAM contraction. In contrast, the rubber rings of the sPAM stretched as applied pressure was increased, inducing an inactive region and causing large deviation from the PPAM model. Maximum BAM contraction was 26.5% at 40 kPa, significantly larger than the sPAM contraction of 18.2% at the same pressure.

### BAM characterization

The BAM introduces new behavior, an overlapped region, which, together with the inactive region present in the sPAM (Fig. 2D), reduces maximum contraction and causes deviation from the PPAM mathematical model. The overlapped region occurs when there is a large amount of folds around the actuator ends, causing crossover of the folds from two ends and overlapped membrane across the actuator. It increases the membrane stiffness and difficulty in unfolding and bending of the folded membrane, and it reduces the bubble radius $R_{bubble}$. This stops the actuator from reaching its optimum shape, leading to partial shape expansion and lower maximum contraction.

**FIG. 7.**
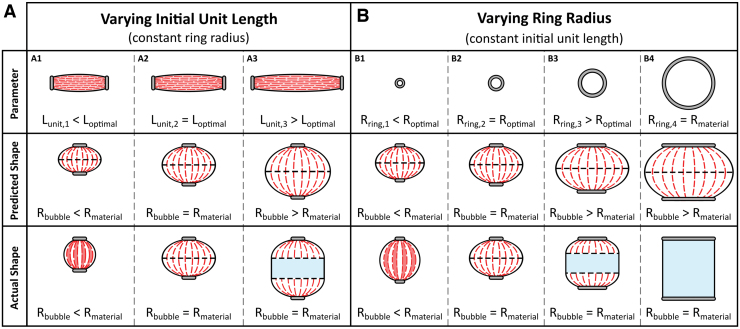
The BAM parameter (*top row*), the predicted PPAM shape (*middle row*), and the actual BAM shape (*bottom row*) at maximum shape expansion when **(A)** varying Lunit with constant Rring and **(B)** varying Rring with constant Lunit. Color images are available online.

When fixing $R_{ring}$ and varying $L_{unit}$ (Fig. 7A), the optimal “bubble” shape with $c_{optimal}$ can be achieved when the BAM possesses $L_{unit}=L_{optimal}$ as shown in Figure 7A, column A2. Decreasing $L_{unit}$ below $L_{optimal}$ ($L_{unit}<L_{optimal}$) causes a partial shape expansion, creating overlapping of the material on the actuator surface, shown as the red area in Figure 7A, column A1. On the other hand, increasing $L_{unit}$ over $L_{optimal}$ ($L_{unit}>L_{optimal}$) causes an inactive region, shown as the blue area in Figure 7A, column A3.

Likewise, the overlapped region and the inactive region occur when varying $R_{ring}$(Fig. 7B). When fixing $L_{unit}$ and increasing $R_{ring}$, the radius of the actuator ends approaches the maximum material radius $R_{material}$, reducing the amount of folding at the actuator ends and the fold length $L_{fold}$ until $L_{fold}=0$ at $R_{ring}=R_{material}$, as shown in columns B1, B2, B3, and B4, respectively (the black dashed line shows the end of the folds). This leads to the inactive region shown in Figure 7B, column B3 and eventually no contraction in column B4. $L_{fold}$ is unaffected by $L_{unit}$ (Fig. 7A, columns A1, A2, and A3).

Although the inactive region can be addressed by decreasing $R_{ring}$, too small $R_{ring}$ can result in the overlapped region as shown in Figure 7B, column B1. The optimal ring radius $R_{optimal}$ will create neither an inactive region nor an overlapped region, and it will deliver the highest contraction (Fig. 7B, column B2). The approach to calculate $L_{optimal}$ and $R_{optimal}$ to design the optimal BAM, which produces $c_{optimal}$, is presented in the BAM Optimal Unit Length and Optimal Ring Radius section in Supplementary Data.

### BAM actuation model

Although the BAM produces lower performance than the PPAM theoretical maximum (Figs. 4 and 5) because of the fundamental difference in their folding structure, they share the same ideal behavior of shape expansion (Fig. 2B). Consequently, the BAM mathematical model is built on the PPAM model with the addition of the inactive region modeled by the sPAM model, and modifications to model the effects of material thickness (Supplementary Figs. S7 and S8). The PPAM and sPAM models overestimate BAM performance in terms of contraction and tensile force, as they do not account for material thickness, a major factor in BAM actuation. This deviation can be reduced by modifying the inelastic PPAM model with an additional term based on experimental observation. The effect of the inactive region from the sPAM model, which can limit the maximum contraction, is also included in the BAM model.

As described earlier in the Varying Material Thickness ($s_{material}$) section and Figure 5B, the ratio of the material thickness and the ring radius ($s_{material}∕R_{ring}$) and the ratio of the material radius and the ring radius ($R_{material}∕R_{ring}$) are likely to be dominant factors in determining the reduction in the BAM performance compared with the PPAM model. As such, the loss in contraction *c* and tensile force *T* can be described by the following equation, where *A* and *n* are constant.

(4) $$loss\propto A*{\left(\frac{s_{material}}{R_{ring}}\cdot \frac{R_{material}}{R_{ring}}\right)}^{n}$$

The relationship between tensile force and contraction of the BAM (Fig. 5A) can be modified from the PPAM model by using the subtractive contraction loss $c_{loss}$, applying from Equation (4). Therefore, the BAM contraction $c_{BAM}$ can be derived from Equation (5), where $c_{PPAM}$ is the PPAM contraction, and where *A* and *n* have been chosen to fit experimental data.

(5) $$\begin{array}{cc} c_{BAM} & =c_{PPAM}-c_{loss}whenc_{loss}\\ & =21.35*{\left(\frac{s_{material}R_{material}}{R_{ring}^{2}}\right)}^{0.25} \end{array}$$

**FIG. 8.**
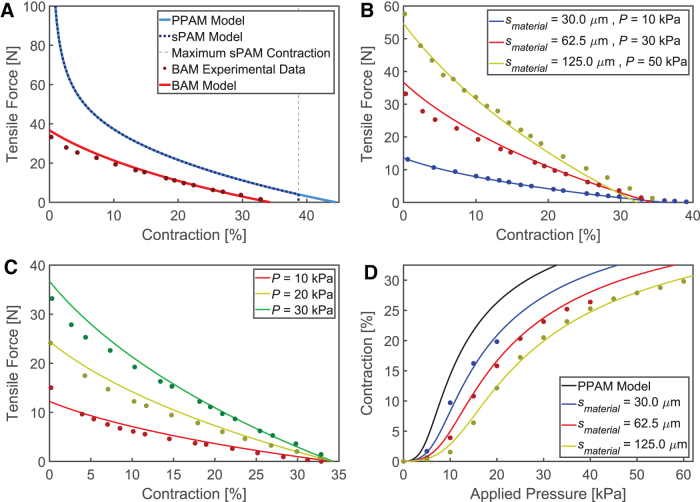
**(A)** Comparison between the PPAM model, sPAM model, and BAM model for a BAM with Lunit = 40.5 mm, Rring = 4.5 mm, and smaterial = 62.5 μm, operated at *P* = 30 kPa, showing the relationship between tensile force and contraction. The maximum sPAM contraction without an inactive region occurring, and the BAM experimental data are included. **(B, C)** BAM model applying subtractive contraction loss (*lines*) and experimental results (*dots*) of BAMs with Lunit = 40.5 mm and Rring = 4.5 mm, showing the relationships between tensile force and contraction when **(B)** varying smaterial and *P* and **(C)** having constant smaterial = 62.5 μm and operating at different *P*. **(D)** BAM model applying multiplicative tension loss (*lines*) and experimental results (*dots*) of BAMs with Lunit = 40.5 mm and Rring = 4.5 mm, showing the relationship between contraction and pressure when varying smaterial and operating at different *P*. Color images are available online.

The comparison between the PPAM model, the sPAM model, the modified model for the BAM, and the experimental data of the BAM ($s_{material}=$ 62.5 μm from Fig. 5A) is presented in Figure 8A. Applying $c_{loss}$ from Equation (5) causes shifting of the PPAM model to match the BAM experimental data (Supplementary Fig. S7). Although not required for the results in Figure 8A, the BAM model also includes the limitation on maximum contraction due to the inactive region, first described in the sPAM model (Supplementary Figs. S7 and S8). The BAM model for the BAMs made of different $s_{material}$ (Fig. 5A) can be seen in Figure 8B. This model can also be used to predict the real-world contraction of the BAM with constant $s_{material}$ at various applied pressures (Fig. 8C).

In contrast, the relationship between contraction and pressure (Fig. 4A) is better modeled by applying a multiplicative tension loss, $T_{loss}=1∕\eta_{loss}$ to PPAM tension $T_{PPAM}$ as in Equation (6). As previously shown experimentally, the BAM requires higher applied pressure *P* than predicted by the PPAM model to deliver desired *T* and *c*. Conversely, this means that $c_{BAM}$ is less than $c_{PPAM}$ at the same *P* and *T*. Figure 8D illustrates the increasing divergences of $c_{BAM}$ from $c_{PPAM}$ with $s_{material}$ while loaded with T= 1 kg.

(6) $$\begin{array}{cc} T_{BAM} & =\frac{1}{\eta_{loss}}*T_{PPAM}when\eta_{loss}\\ & =4.39*{\left(\frac{s_{material}R_{material}}{R_{ring}^{2}}\right)}^{0.31} \end{array}$$

### Design optimization

**FIG. 9.**
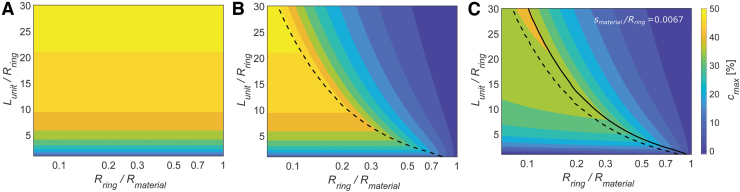
Model-predicted maximum contraction of **(A)** PPAM, **(B)** sPAM, and **(C)** BAM made of smaterial = 30.0 μm. *Color* shows the maximum contraction cmax of the actuators as Lunit/Rring and Rring/Rmaterial are varied. For each Rring/Rmaterial, *dashed* and *solid lines* indicate the location where maximum contraction is highest based on the sPAM and BAM model, respectively. Color images are available online.

Analysis summarized in Figure 9 shows how the BAM actuator differs from the PPAM and the sPAM, and how it can be optimized to achieve high contraction by choosing three ratios of material properties: $L_{unit}∕R_{ring}$, $R_{ring}∕R_{material}$, and $s_{material}∕R_{ring}$. Figure 9A shows the maximum contraction predicted by the PPAM model, simulated by substituting $L_{unit}∕R_{ring}$ in Equations (1) and (2). It does not show an overlapped region or an inactive region since the PPAM actuator membrane is uniformly folded and its actuation never reaches maximum shape expansion, and its contraction is maximized as $L_{unit}∕R_{ring}$ and does not depend on $R_{ring}∕R_{material}$.

Figure 9B shows the maximum contraction predicted by the sPAM model. At any $R_{ring}∕R_{material}$, contraction increases with $L_{unit}∕R_{ring}$ until reaching a maximum when $L_{unit}=L_{optimal}$. If $L_{unit}∕R_{ring}$ is further increased, contraction reduces due to the inactive region. Neither the PPAM nor the sPAM models account for the effect of $s_{material}$, which is a significant factor influencing the contractile performance. Figure 9C shows the BAM model presented in this article; the effect of material thickness slightly reduces maximum contraction, and it slightly increases the optimal $L_{unit}∕R_{ring}$ at each $R_{ring}∕R_{material}$ compared with the sPAM model. The optimal design of the BAM made of different $s_{material}$ is shown in Supplementary Figure S9.

### BAM improvement

The performance of the BAM can be improved by several approaches. First, better quality fabrication and a method for creating uniformly lateral folding along the actuator are required so that the BAM can unfold and expand more easily with lower friction. Uniform folding can increase BAM contraction and decrease the deviation between the mathematical model and the practical performance.

For the current design, the rings are placed without any attachments to the tubing. After repeated actuation, the rings tend to be fixed in place by the shape adopted by the folded membrane; as $R_{material}$ is much larger than $R_{ring}$, the folded membrane passively bends around the ring, helping to constrain the movement of the rings. If BAM actuators are to be integrated into a robotic system without prior actuation, the rings can be fixed in place by adhesive to prevent slippage. Alternatively, one future BAM design could have no rings but would use its own actuator membrane to form the “bubble” shape, for example, by applying origami or kirigami methods so that the bubble shape naturally emerges.

Last, using high-strength inelastic materials, the BAM can operate under higher applied pressure to deliver higher tensile force. The ideal actuator membrane should possess high Young's Modulus and extremely high tensile strength to withstand high pressure for high tensile force, have low interfacial friction, and be very thin and very flexible to expand easily at low-pressure actuation and deliver high contraction.

## BAM Orthosis for Sit-to-Stand Transition

**FIG. 10.**
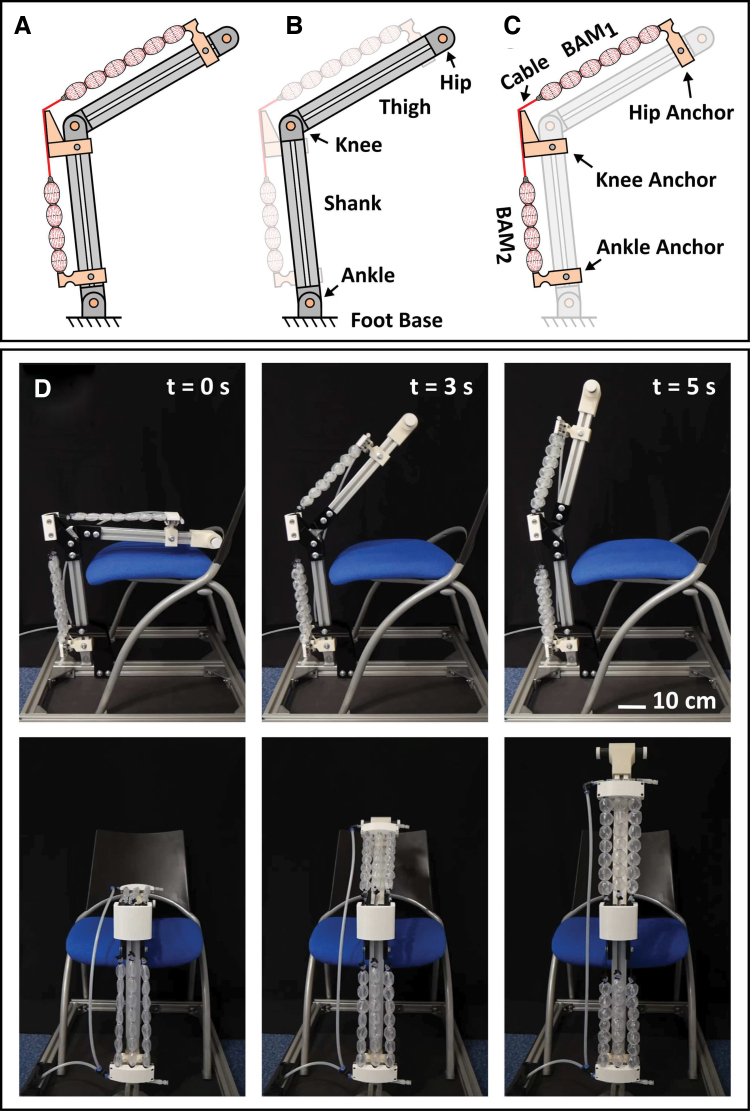
The experimental setup of the sit-to-stand motion **(A)** is composed of two main parts: the leg mechanism **(B)** and the BAM orthosis **(C)**. **(D)** Actuation of the BAM orthosis on the human-like leg mechanism to perform standing motion, operated at 50 kPa. Color images are available online.

A leg mechanism was built to evaluate the BAM performance in assisting human mobility in the task of standing up from a sitting position (sit-to-stand), as shown in Figure 10A. It consists of three segments: a foot base, a shank rod, and a thigh rod, which are connected via revolute joints representing the knee and ankle (Fig. 10B). It was designed so that the shank rod can be fixed at an adjustable ankle angle and an external load can be added to the hip joint, to represent a body weight. A BAM orthosis was created by using three pairs of BAMs aligned in parallel (Fig. 10D). In each pair, BAMs are connected in series by cables and located at the thigh and shank at 7.5 cm from the center axis, labeled as BAM_1_ and BAM_2_, respectively (Fig. 10C).

Each BAM contains seven contractile units, where $L_{actuator}$ = 305.0 mm, $L_{unit}$ = 40.5 mm, $R_{ring}$ = 4.5 mm, and $s_{material}$ = 125.0 μm. The ends of BAM_1_ and BAM_2_ connect to the hip and ankle anchors and are joined by connecting cables that pass through the knee anchor. Based on the maximum tensile force of a single BAM of 56.9 N, the effective support force for the sit-to-stand transition was 171 N. The BAM orthosis was able to perform sit-to-stand transition within 5 s, supplied by the air compressor at *P* = 50 kPa (Fig. 10D). The device could be attached to a human subject by using tight straps, or it could be attached to a passive orthotic such as a knee-brace to convert it to an assistive device.

## Conclusion

The BAM is one of the most lightweight pneumatic actuators (<6 g) that can deliver either high contraction or high tension depending on the actuator's size and thickness and the stiffness of the constituent material. A thicker actuator membrane allows the BAM to operate under higher applied pressure to produce higher tensile force, whereas metal rings can maintain the actuator radius, delivering desired contraction when operated at high pressure. Despite having a different folding pattern to the PPAM, the BAM can form a similar inflated shape and function but with considerably simpler and low-cost fabrication. The optimal BAM can be achieved by choosing the unit length and ring radius to suit the material radius and thickness, avoiding both an overlapped region and an inactive region.

The BAM actuation model was built based on the PPAM model with an additional loss term, modifying the model to match the BAM experimental results. This model can predict the real-world performance for BAMs made from different material thicknesses and operated at different applied pressures. In this work, the loss term was empirically derived, and it demonstrates how BAM and sPAM behavior differ and how BAM performance can be improved. In the future, we plan to derive a BAM loss term from first principles and compare this theoretical model with the empirically derived model.

The BAM was designed to interact with the human body in the form of a wearable exosuit or orthosis. It fulfills this design brief by being lightweight and scalable, and by generating human-scale forces and contractions at low pressure. An example of sit-to-stand transition was demonstrated by using a leg mechanism assisted by a BAM orthosis, exhibiting sit-to-stand within 5 s. The BAM can be improved further by exploiting actuator materials that possess high strength and flexibility to achieve higher tension and contraction, removing the requirement of retaining rings by using its own material as the structure to form a contractile shape, and improving fabrication quality to create uniform folding for lower-friction shape expansion.

## Supplementary Material

Supplemental data

Supplemental data

## Data Access Statement

Data necessary to support the conclusions are included in the article.
