# Supplementary material for: Characteristic Analysis and Design Optimization of Bubble Artificial Muscles
Source: Soft Robot. 2021 Apr 16;8(2):186–99. doi: 10.1089/soro.2019.0157 (PMC8082735; doi:10.1089/soro.2019.0157)
Supplement: Supplemental data [file Supp_Data.pdf]

Supplementary Data

Pleated Pneumatic Artificial Muscles

The pleated pneumatic artificial muscle (PPAM) is made of a polymer with high tensile strength and high flexibility (actuator membrane—pink in Supplementary Fig. S1A) connected to two end fittings (actuator ends—gray in Supplementary Fig. S1A). The end fittings feature several radial teeth, which create a series of pleats with equal spacing around the actuator end (cross-section i in Supplementary Fig. S1A). When inflated, these pleats unfold, allowing the actuator membrane to expand circumferentially and radially, reaching a maximum inflated radius at the actuator center (see cross-sections i, ii, iii, and iv). Unfolding of these pleats happens with negligible friction and energy loss. The actuator's shape and its contraction are defined by m, as demonstrated in Figure 2B. For example, an actuator at zero contraction ($c = 0$, blue solid line in Fig. 2B) has an m value equal to 0, whereas the actuator at maximum contraction ($c = c_{max}$, red dotted line in Fig. 2B) has an m value equal to 0.5.

Elliptic integral

Modeling the PPAM requires an elliptic integral of the first kind $F(\varphi/m)$ and second kind $E(\varphi/m)$ as follows. These equations can be solved with the use of MATLAB (a numerical computing program):

$$F(\varphi/m) = \int_0^{\varphi} \frac{1}{\sqrt{1 - msin^2\theta}} d\theta \qquad \text{(S1)}$$

$$E(\varphi/m) = \int_0^{\varphi} \sqrt{1 - msin^2\theta} d\theta \qquad \text{(S2)}$$

PPAM mathematical model

According to the PPAM model presented in the Pleated Pneumatic Artificial Muscle section, three main Equations (1–3) are used to calculate the contraction c and tensile force T of the PPAM with the use of the elliptical integral with dominant parameters, m and φ_R. This model assumes inelastic behavior of the actual material. The derivation of those equations of the PPAM mathematical model was summarized from Daerden[S1] and is shown in the following sections.

The actuator shape at given contraction is defined by x_i and r_i ($i = 0, 1, \ldots, n$), representing a point on the actuator surface as a horizontal axial distance from the actuator center and a membrane radius from the axial line, respectively (Supplementary Fig. S1C). Each point on the actuator surface can be acquired by solving Equations (S3) and (S4) with constant R, m, and φ_R, and different φ, varying from 0 to φ_R. For instance, when $\varphi = \varphi_R$, (x_i, r_i) is equal to (x_0, r_0), where x_0 is at the actuator end and $r_0 = R$. When $\varphi = 0$, (x_i, r_i) is equal to (x_n, r_n), where x_n is at the actuator center ($x_n = 0$) and $r_n = r_{max}$.

$$x_i = \frac{R}{\sqrt{m}\cos\varphi_R}\left(E(\varphi/m) - \frac{1}{2}F(\varphi/m)\right) \qquad \text{(S3)}$$

$$r_i = \frac{R}{\cos\varphi_R}\cos\varphi \qquad \text{(S4)}$$

where $F(\varphi/m)$ and $E(\varphi/m)$ are the functions of the elliptic integral of the first kind and second kind (Elliptic Integral section), and m and φ_R are parameters defining the shape of the actuator. In addition to the actuator shape, the maximum actuator radius R_{max} at the actuator center and the actuator volume V at any contraction c can be calculated by using the following equations:

$$R_{max} = \frac{R}{\cos\varphi_R} \qquad \text{(S5)}$$

$$V = \frac{\pi R^2 L}{6m\cos^2\varphi_R}\left(1 - (1-2m)(1-c) + \frac{2R}{L}\sqrt{1 - \left(1 - 2m\sin^2\varphi_R\right)^2}\right) \qquad \text{(S6)}$$

Angle φ_R. Considering the actual membrane length of the actuator L_{actual} when dL is a small length along the actuator surface from $-x_0$ to x_0 (Supplementary Fig. S1C),

$$L_{actual} = \int_{-x_0}^{x_0} dL$$

The integration can be solved with the use of the elliptical integral.[S1] Therefore,

$$L_{actual} = \frac{R}{\sqrt{m}\cos\varphi_R}F(\varphi_R/m) \qquad \text{(S7)}$$

Assuming that L_{actual} is different from the resting actuator length L by a membrane strain ε caused by a meridional stress under an applied pressure P,

$$\varepsilon = \frac{L_{actual} - L}{L}$$

$$L_{actual} = L(1 + \varepsilon)$$

The strain ε can be converted to a function of a, m, and φ_R.[S1] Therefore,

$$L_{actual} = L\left(1 + \frac{a}{2mcos^2\varphi_R}\right) \qquad \text{(S8)}$$

where a implies the elasticity of the actuator's membrane, which is a function of an applied pressure P, an actuator radius R, a cross-sectional area at the actuator end A, and Young's Modulus E of the actuator's membrane as follows:

$$a = \frac{\pi P R^2}{AE}$$

From Equations (S7) and (S8),

$$\frac{R}{\sqrt{m}\cos\varphi_R}F(\varphi_R/m) = L\left(1 + \frac{a}{2mcos^2\varphi_R}\right) \quad \text{(S9)}$$

Equation (S9) is used to calculate the angle φ_R of the PPAM with elasticity assumption of the actuator's membrane. When assuming that the membrane is inelastic, a becomes 0. Therefore,

$$L = \frac{R}{\sqrt{m}\cos\varphi_R}F(\varphi_R/m) \quad (1)$$

Contraction *c*. Consider the contraction c in the axial direction of the actuator when L is the actuator length and x_0 is half of the axial actuator length when the actuator is inflated (Supplementary Fig. S1). Therefore,

$$c = \frac{L - 2x_0}{L}$$

$$x_0 = \frac{L}{2}(1 - c) \quad \text{(S10)}$$

x_0 can be derived by the elliptical integral[S1] as follows:

$$x_0 = \frac{R}{\sqrt{m}\cos\varphi_R}\left(E(\varphi_R/m) - \frac{1}{2}F(\varphi_R/m)\right) \quad \text{(S11)}$$

From Equations (S10) and (S11),

$$\frac{L}{2}(1 - c) = \frac{R}{\sqrt{m}\cos\varphi_R}\left(E(\varphi_R/m) - \frac{1}{2}F(\varphi_R/m)\right)$$

Therefore, the contraction c can be derived as follows:

$$c = 1 - \frac{2R}{L}\left(\frac{E(\varphi_R/m) - \frac{1}{2}F(\varphi_R/m)}{\sqrt{m}\cos\varphi_R}\right) \quad (2)$$

Tensile force T

PPAM Tension T at a given contraction, derived from a pair of m and φ_R, can be calculated when knowing R and P[S1] as follows:

$$T = \pi P R^2 \frac{1 - 2m}{2m\cos^2\varphi_R} \quad (3)$$

According to the PPAM, when having constant pressure $P_{constant}$, the predicted tension to achieve a given contraction [from Equation (2) with a pair of m and φ_R value] can be calculated as follows:

$$T = \pi P_{constant} R^2 \frac{1 - 2m}{2m\cos^2\varphi_R} \quad \text{(S12)}$$

In contrast, when having constant tension $T_{constant}$, the predicted pressure to achieve a given contraction can be calculated as follows:

$$P = \frac{T_{constant}}{\pi R^2} \cdot \frac{2m\cos^2\varphi_R}{1 - 2m} \quad \text{(S13)}$$

Summary of the PPAM mathematical model

The PPAM mathematical model can be solved to obtain contraction and tensile force (for a given pressure) or applied pressure (for a given tensile force) as summarized in Supplementary Figure S2; the actuator length L and actuator radius R are initial parameters. The bubble artificial muscle (BAM) applies this mathematical model by substituting an initial unit length L_{unit} and a ring radius R_{ring} instead of L and R.

BAM Optimal Unit Length and Optimal Ring Radius

The optimal BAM is the BAM with either optimal unit length $L_{optimal}$ or optimal ring radius $R_{optimal}$, forming the maximum expanding shape ($m = 0.5$) resembling a bubble (when $R_{bubble} = R_{material}$ or $L_{bubble} = 2L_{fold}$) and achieving $c_{optimal}$ without the existence of an overlapped region or an inactive region as shown in Figure 7 (columns A2 and B2). For the optimal BAM, $L_{optimal}$ and $c_{optimal}$ can be calculated by first selecting R_{ring} and $R_{material}$ of the BAM and using Equations (S5) and (1) as follows:

First, calculate $\varphi_{R,L_{optimal}}$ when $R_{bubble} = R_{material}$ using Equation (S5),

$$R_{bubble} = R_{material} = \frac{R_{ring}}{\cos\varphi_{R,L_{optimal}}}$$

Therefore,

$$\varphi_{R,L_{optimal}} = \cos^{-1}\left(\frac{R_{ring}}{R_{material}}\right) \quad \text{(S14)}$$

Substituting R_{ring}, $\varphi_{R,L_{optimal}}$ [calculated from Equation (S14)], and $m = 0.5$ (maximum shape expansion) in Equation (1) to obtain $L_{optimal}$,

$$L_{optimal} = \frac{R_{ring}}{\sqrt{m}\cos\varphi_{R,L_{optimal}}}F\left(\varphi_{R,L_{optimal}}/m\right)$$

This equation can also be considered as the ratio of $L_{optimal}/R_{ring}$ in the following equation:

$$\frac{L_{optimal}}{R_{ring}} = \frac{F\left(\varphi_{R,L_{optimal}}/m\right)}{\sqrt{m}\cos\varphi_{R,L_{optimal}}} \quad \text{(S15)}$$

$c_{optimal}$ can be derived by using selected R_{ring}, calculated $L_{optimal}$ and $\varphi_{R,L_{optimal}}$, and $m = 0.5$ with Equation (2):

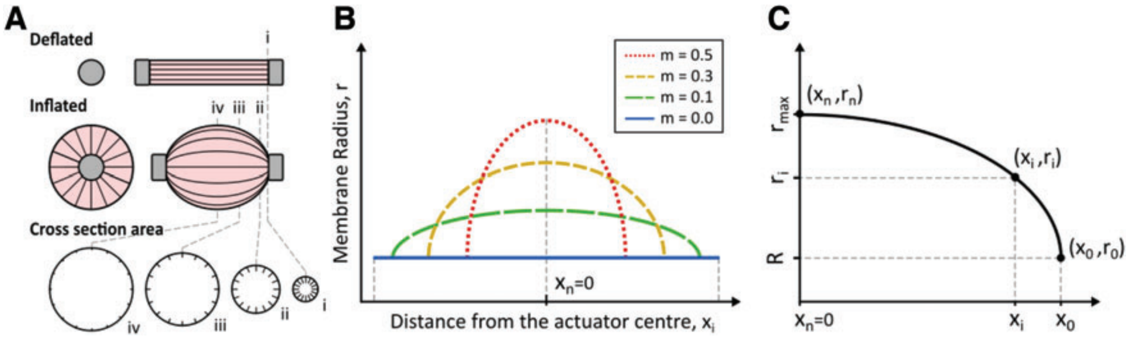

SUPPLEMENTARY FIG. S1. **(A)** The deflated and inflated shapes of the PPAM in *top* and *side* views with selected cross-sections perpendicular to the actuator axis. **(B)** Profile of the PPAM at different contractions simulated by using different m values. **(C)** The definition of the parameters on the membrane surface, x and r, as a distance from the actuator center and a membrane radius. Modified and adapted from the PPAM model.[S1] PPAM, pleated pneumatic artificial muscle.

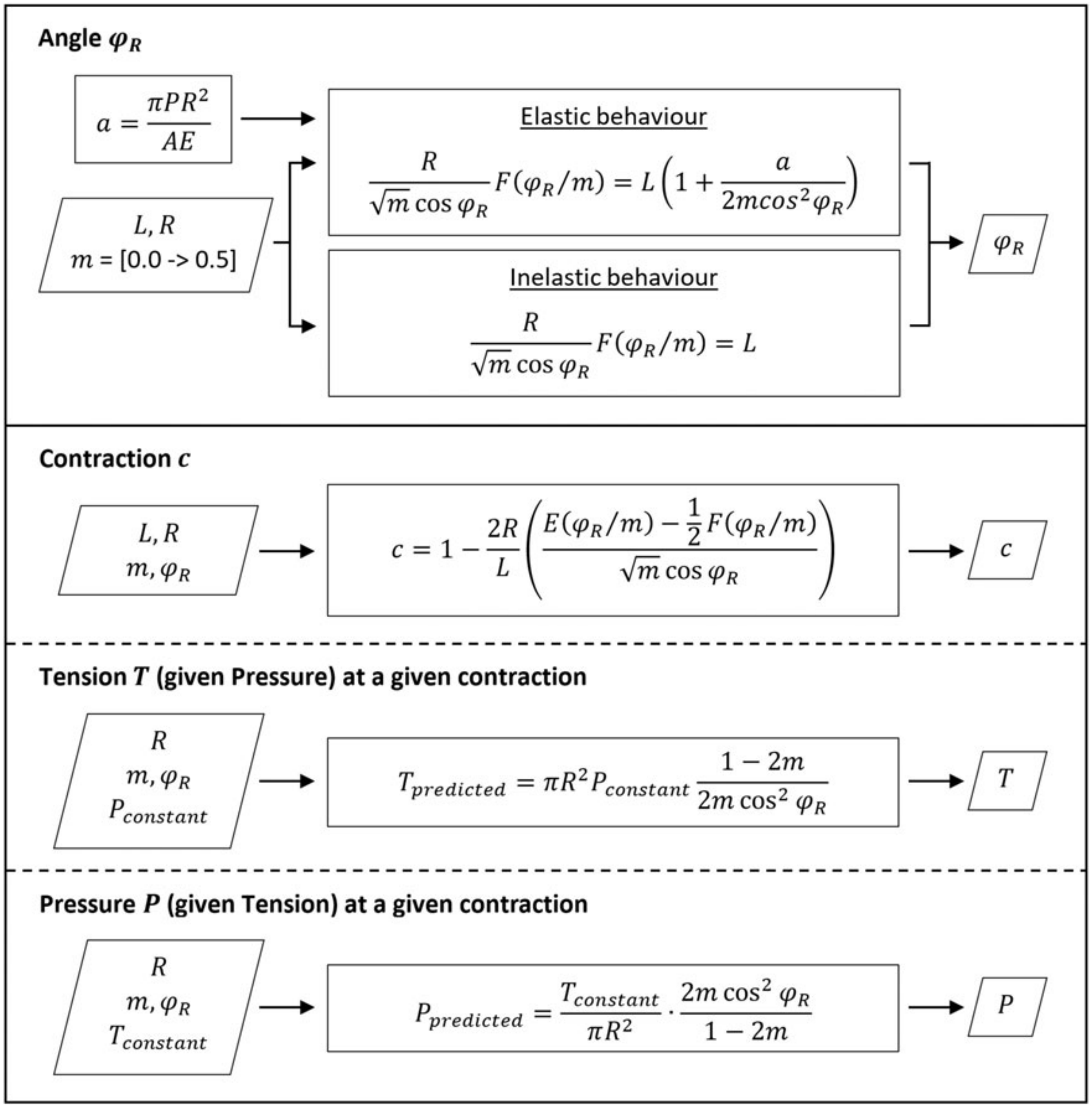

SUPPLEMENTARY FIG. S2. Summary of the PPAM mathematical model with input and output parameters.

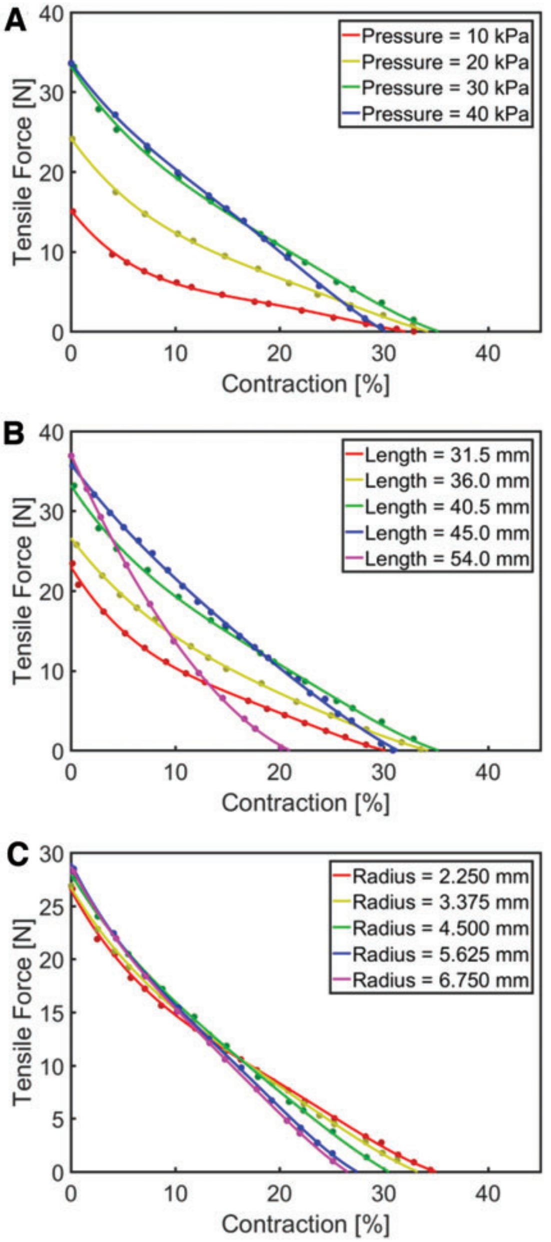

SUPPLEMENTARY FIG. S3. The relationship between tensile force and contraction of BAMs acquired from the investigation of the effect of **(A)** increasing applied pressure, **(B)** varying initial unit length, and **(C)** varying ring radius at any stroke ΔL when other parameters are constant ($L_{unit} = 40.5$ mm, $R_{ring} = 4.5$ mm, and $P = 30$ kPa). The *dots* and *lines* represent experimental data and fitting curves, respectively. BAMs, bubble artificial muscles.

$$c_{optimal} = 1 - \frac{2R_{ring}}{L_{optimal}}\left(\frac{E\left(\varphi_{R,L_{optimal}}/m\right) - \frac{1}{2}F\left(\varphi_{R,L_{optimal}}/m\right)}{\sqrt{m}\cos\varphi_{R,L_{optimal}}}\right) \tag{S16}$$

Alternatively, the optimal BAM with $R_{optimal}$ can be created when initially selecting L_{unit} and $R_{material}$ to calculate $R_{optimal}$. First, using an unknown $R_{optimal}$ instead of R_{ring} in Equation (S14) as follows,

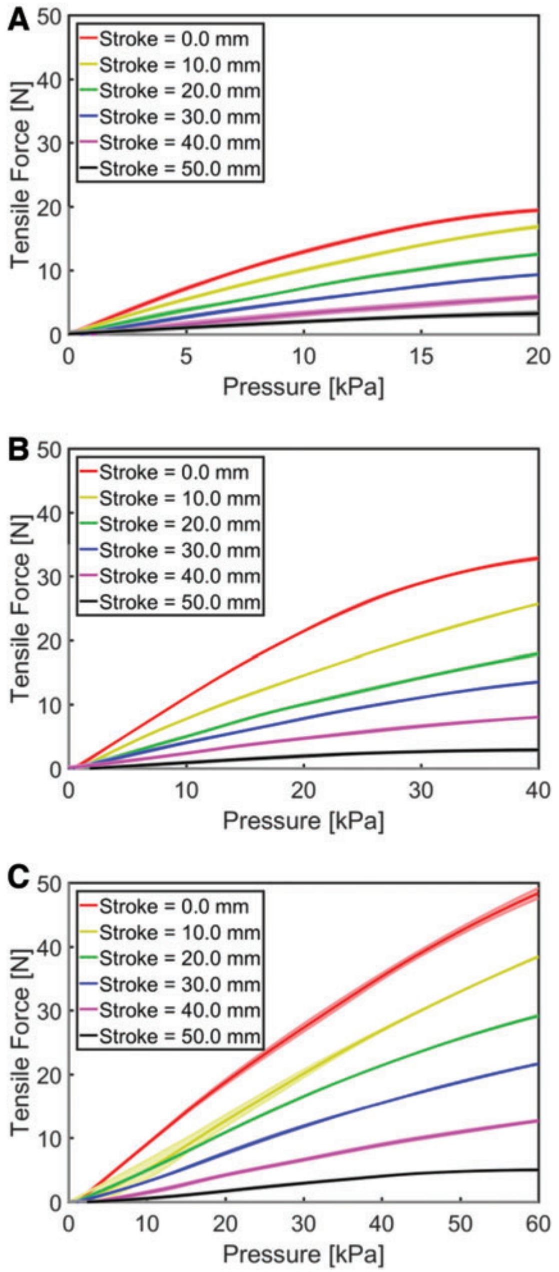

SUPPLEMENTARY FIG. S4. The relationship between tensile force and pressure of BAMs with $L_{unit} = 40.5$ mm, $R_{ring} = 4.5$ mm, and different $s_{material} = 30.0\,\mu$m **(A)**, 62.5 μm **(B)** and 125.0 μm **(C)** from isometric tests, when fixing the actuator at different stroke (ΔL) values. Different-thickness BAMs are inflated to a different maximum pressure. The *solid line* is the mean of the three trials for each condition, and the faded area defines the total range (minimum to maximum) of the experimental results.

$$\varphi_{R,R_{optimal}} = \cos^{-1}\left(\frac{R_{optimal}}{R_{material}}\right) \tag{S17}$$

Rearranging Equation (1) to calculate $R_{optimal}$,

$$R_{optimal} = \frac{L_{unit}}{F\left(\varphi_{R,R_{optimal}}/m\right)}\sqrt{m}\cos\varphi_{R,R_{optimal}} \tag{S18}$$

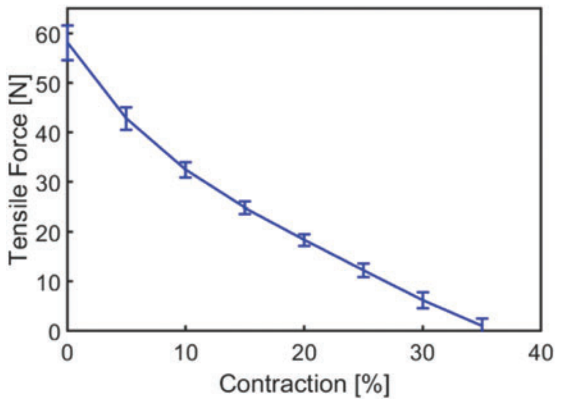

SUPPLEMENTARY FIG. S5. Sample variation of three BAMs, made of the same $s_{material} = 125.0\,\mu$m, $L_{unit} = 40.5$ mm, and $R_{ring} = 4.5$ mm, actuated at $P = 50$ kPa, showing tension–contraction relationship.

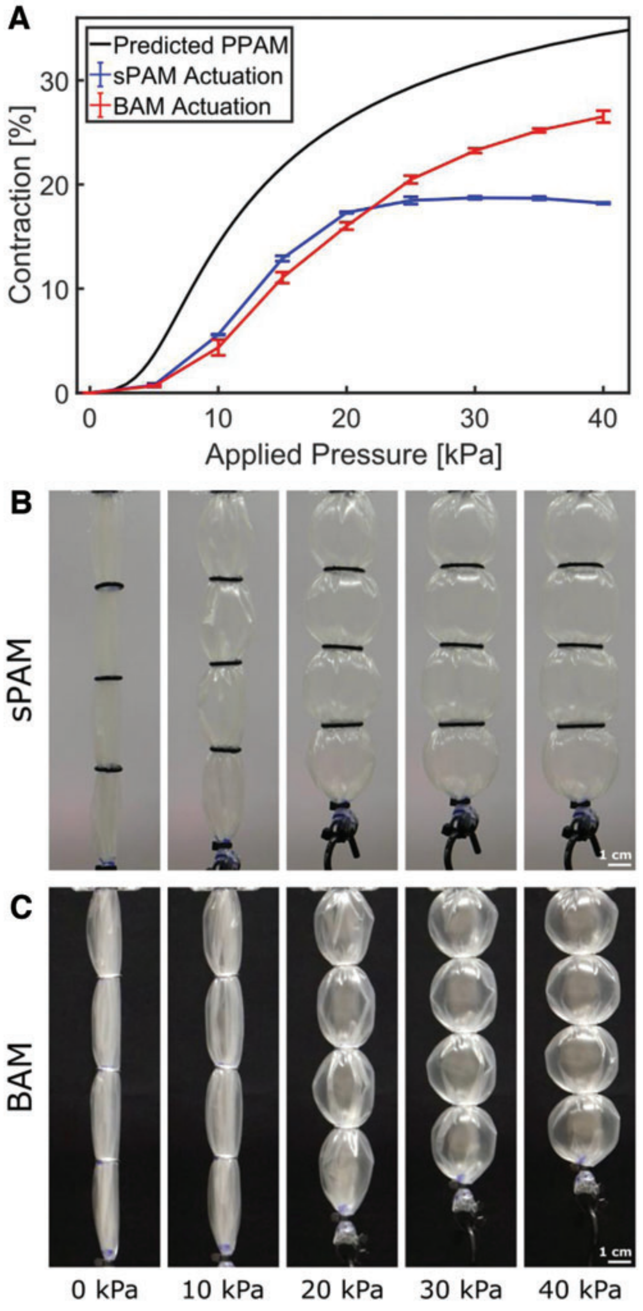

SUPPLEMENTARY FIG. S6. **(A)** Comparison between theoretical PPAM contraction, actual sPAM contraction, and actual BAM contraction, during an isotonic test with a load of 1 kg. Both the sPAM **(B)** and BAM **(C)** were made of plastic tubing with $s_{material} = 62.5\,\mu$m and $L_{unit} = 40.5$ mm, and rubber rings (sPAM) or metal retaining rings (BAM) with $R_{ring} = 4.5$ mm. The sPAM rubber rings had a ring thickness of 2 mm, whereas the BAM metal rings had a ring thickness of 1.3 mm. sPAM, series pneumatic artificial muscle.

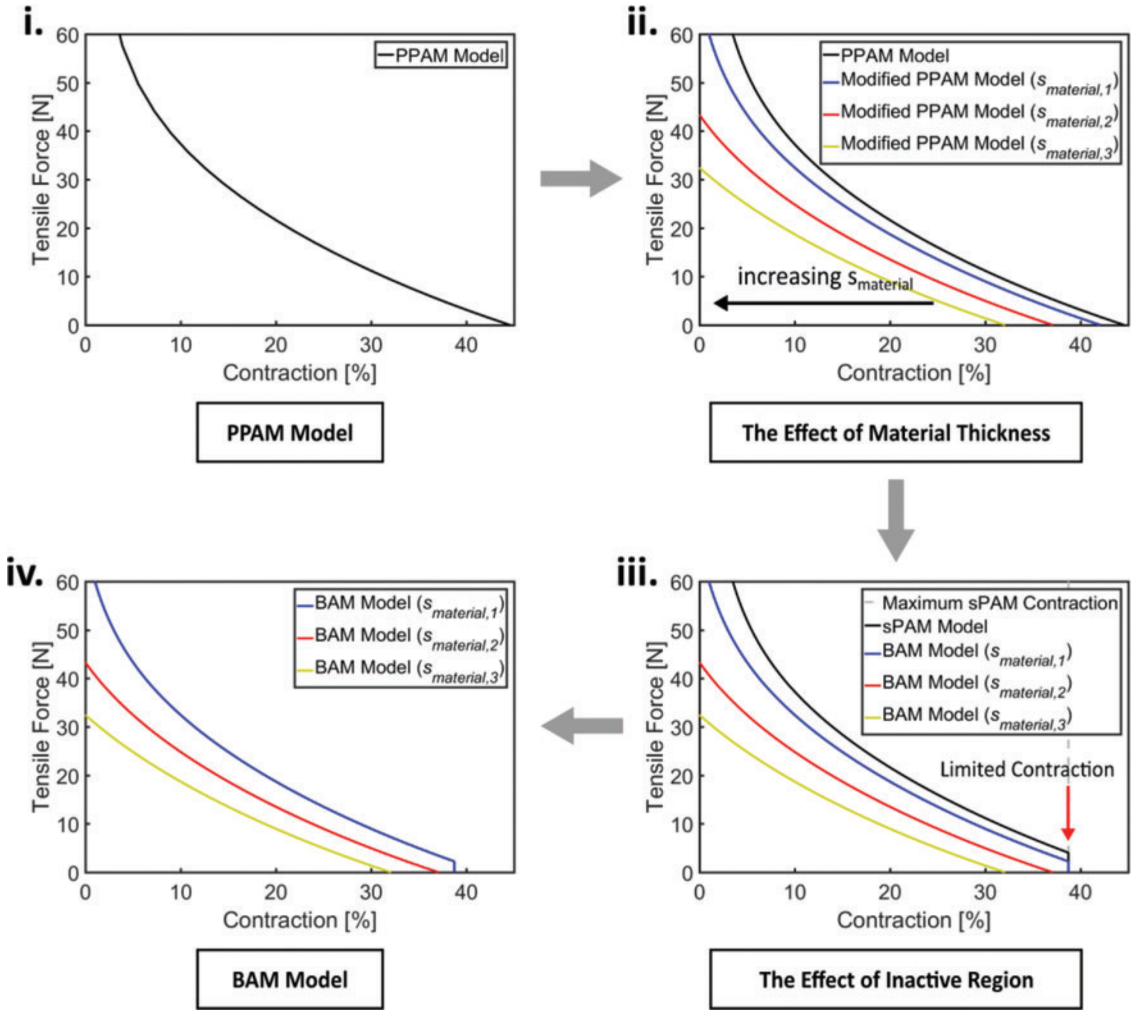

SUPPLEMENTARY FIG. S7. Summary of the BAM model: **(i)** calculate the relationship between tensile force and contraction from the PPAM model. **(ii)** Apply the effect of material thickness ($s_{material,1} < s_{material,2} < s_{material,3}$). **(iii)** Apply the effect of the inactive region, limiting the maximum contraction. **(iv)** The resultant BAM model.

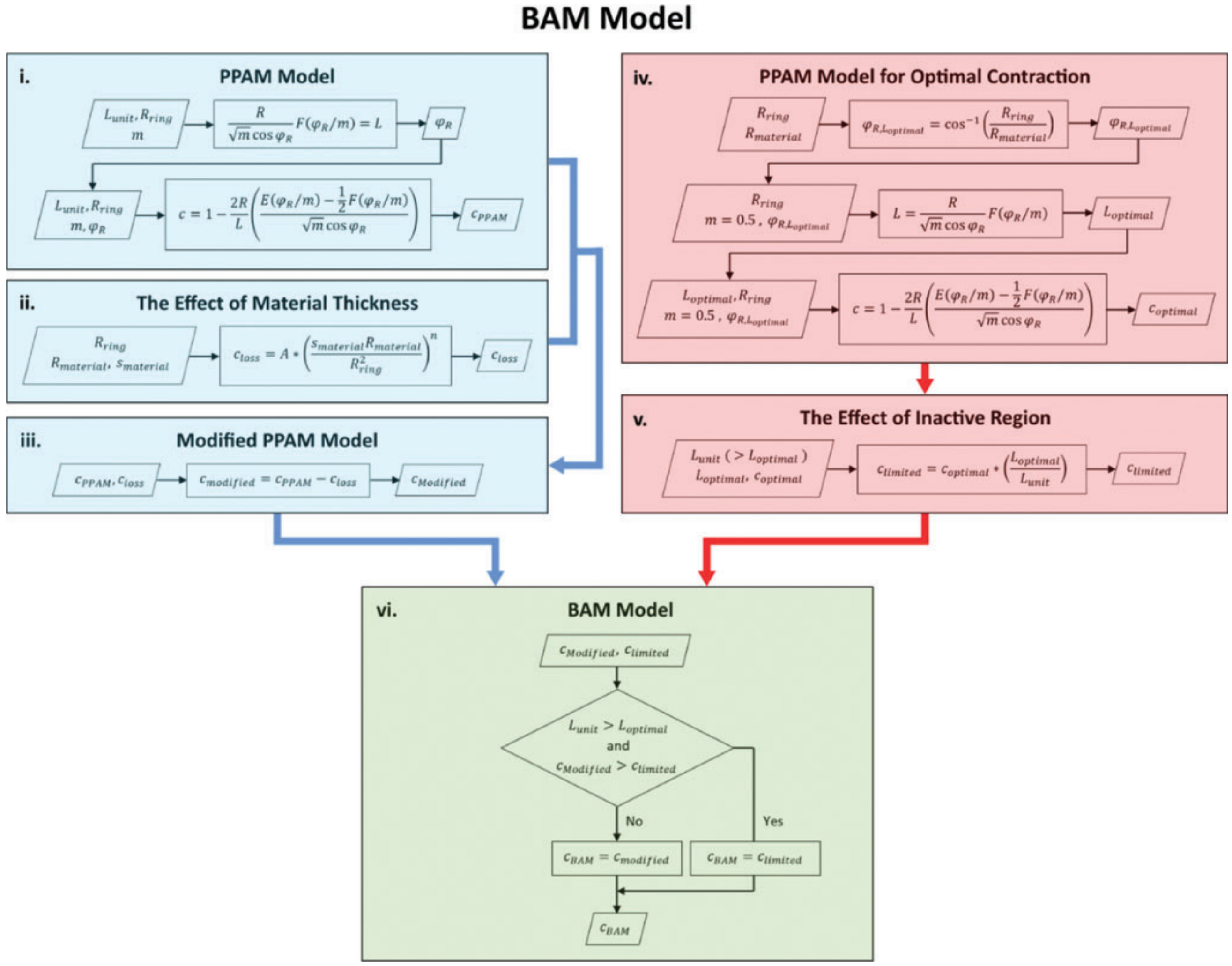

SUPPLEMENTARY FIG. S8. Flow chart of the BAM model for the relationship between tensile force and contraction, calculating BAM contraction c_{BAM}. Tensile force T can be calculated from the PPAM model. **i** to **vi** indicate sequent steps in modelling BAM contraction, calculating: **(i–iii)** modified PPAM contraction using the applied PPAM model and the effect of material thickness, **(iv–v)** optimal contraction and limited contraction due to the inactive region, and **(vi)** BAM contraction.

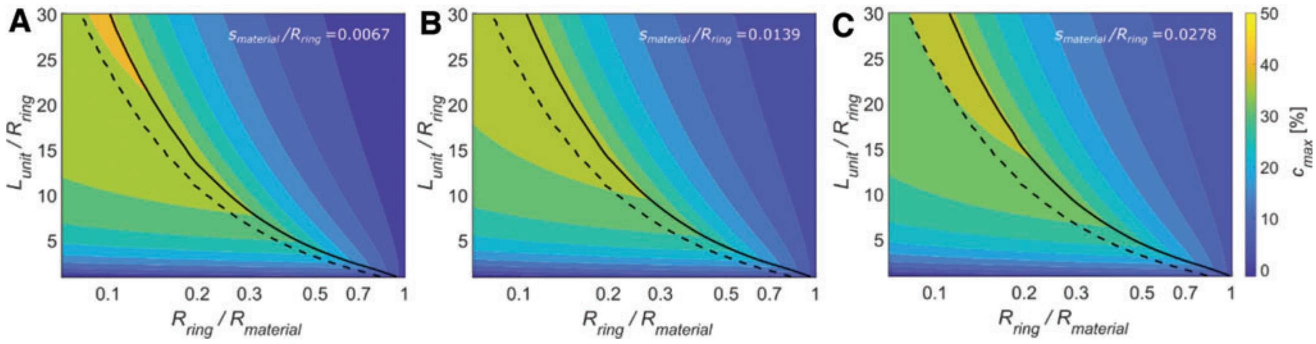

SUPPLEMENTARY FIG. S9. – Model-predicted maximum contraction for BAM made of different $s_{material}$: **(A)** 30.0 μm, **(B)** 62.5 μm, and **(C)** 125.0 μm. *Color bar* shows the maximum contraction c_{max} of actuators as L_{unit}/R_{ring} and $R_{ring}/R_{material}$ are varied. For each $R_{ring}/R_{material}$, *dashed* and *solid lines* indicate the location where maximum contraction is highest based on the sPAM and BAM model, respectively.

Supplementary Table S1. Specifications of Three Selected Low-Density Polyethylene Layflat Tubes Used to Fabricate Bubble Artificial Muscles

Material thickness $s_{material}$ *(μm)*	*Material radius* $R_{material}$ *(mm)*
30.0	16.9
62.5	17.2
125.0	17.8

Substituting $\varphi_{R,R_{optimal}}$ as a function of an unknown $R_{optimal}$ in Equation (S18) and using MATLAB results in the optimal ring radius $R_{optimal}$. This approach is more complicated than the former one since Equations (S17) and (S18) are interdependent. Subsequently, the optimal contraction $c_{optimal}$ of the BAM with $R_{optimal}$ can be calculated from Equation (2) by using L_{unit}, $R_{optimal}$, $\varphi_{R,R_{optimal}}$, and $m = 0.5$. As a result, these two methods ($L_{optimal}$ and $R_{optimal}$) allow us to design the optimal BAM when knowing $R_{material}$ and constraining either only L_{unit} or R_{ring}.

Supplementary Table S2. Theoretical Maximum Zero-Tension Contraction of the Pleated Pneumatic Artificial Muscle and Series Pneumatic Artificial Muscle with Constant

		At $L_{optimal}$	*At* $L_{unit} = 40.5$ *mm*	
R_{ring}	$L_{optimal}$ *(mm)*	c_{PPAM} *(%)*	c_{PPAM} *(%)*	c_{sPAM} *(%)*
2.250	40.6	49.3	49.3*	49.3*
3.375	38.3	46.5	46.9	44.0
4.500	36.1	43.5	44.6	38.7
5.625	33.8	40.3	42.4	33.6
6.750	31.5	36.9	40.2	28.7

$L_{unit} = 40.5$ mm, but various R_{ring}, where $L_{optimal}$ is based on $s_{material} = 62.5$ μm and $R_{material} = 17.2$ mm.

*Shows that $c_{sPAM} = c_{PPAM}$ since $L_{unit} < L_{optimal}$.

SUPPLEMENTARY MOVIE S1. Demonstration of BAM actuation and BAM orthosis for sit-to-stand transition.

Supplementary Reference

S1. Daerden F. Conception and realization of pleated pneumatic artificial muscles and their use as compliant actuation elements. PhD thesis, Vrije Universiteit Brussel, Brussels, 1999;176.
